# Influence of Water Limitation on Morphological, Physiological, and Anatomical Adaptations in Water Hyssop (*Bacopa monnieri* (L.) Wettst.)

**DOI:** 10.3390/plants15213424

**Published:** 2026-11-03

**Authors:** Pattama Tongkok, Gadewara Matmarurat, Pimpilai Saengmanee, Nattanan Panjaworayan T-Thienprasert, Wannarat Phonphoem, Sirinun Suriwong, Chatcharee Kaewsuralikhit

**Affiliations:** 1 Department of Fishery Biology, Faculty of Fisheries, Kasetsart University, 50 Ladyao, Chatuchak, Bangkok 10900, Thailandchatcharee.s@ku.th (C.K.); 2 Kasetsart Agricultural and Agro-Industrial Product Improvement Institute, Kasetsart University, 50 Ladyao, Chatuchak, Bangkok 10900, Thailand; aapgam@ku.ac.th (G.M.); pimpilai.sae@ku.th (P.S.); 3 Department of Biochemistry, Faculty of Science, Kasetsart University, 50 Ladyao, Chatuchak, Bangkok 10900, Thailand; fscinnp@ku.ac.th (N.P.T.-T.); wannarat.p@ku.ac.th (W.P.)

**Keywords:** aquatic medicinal plant, drought stress, phenolic compounds, soil moisture, water hyssop

## Abstract

Water hyssop (*Bacopa monnieri* (L.) Wettst.) is a water-demanding medicinal plant whose responses to declining soil moisture remain insufficiently characterized, particularly in relation to phenolic accumulation. This study investigated the morphological, physiological, and anatomical responses of water hyssop to water limitation and identified the soil moisture conditions associated with enhanced phenolic accumulation. Short-term water limitation (11 days at 9.06% GWC) coincided with peak total phenolic content (TPC), reaching 19.57 mg GAE g^−1^ DW. Physiological stress was initially detected at 21–22% GWC, evidenced by a decline in relative water content (RWC). Furthermore, 8–9% GWC soil moisture marked a critical physiological threshold characterized by a pronounced reduction in photosynthetic performance and elevated oxidative stress markers. Prolonged water limitation (4 weeks at 20–30% GWC) was associated with lower leaf greenness, RWC, and photosynthetic performance. Concurrently, water restriction led to distinct anatomical alterations, including reduced chloroplast abundance, starch accumulation in the stem cortex, and a reduction in stem xylem vessel diameter. These findings indicate that water deficit affects physiological adaptations, accompanied by increased bioactive compound accumulation.

## 1. Introduction

Water hyssop (*Bacopa monnieri* (L.) Wettst.) is an aquatic medicinal plant that has received considerable attention because of its diverse array of bioactive compounds, with potential applications in pharmaceutical and health-related products. Previous studies have demonstrated that this plant has antioxidant and anti-inflammatory properties, and it is used to enhance memory and treat Alzheimer’s and Parkinson’s diseases [1,2,3]. It can also synthesize various chemical compounds, among which triterpenoid saponins, particularly bacosides, are the major bioactive constituents and have been extensively investigated for their pharmacological effects, especially those associated with neurological functions [4,5,6]. In addition to bacosides, water hyssop contains other secondary metabolites with potential biological activities, particularly phenolic compounds and flavonoids, which have been reported to exhibit antioxidant activity and low cytotoxicity. Moreover, polyphenol-rich extracts of water hyssop have demonstrated wound-healing activity [7,8,9]. Several phenolic compounds, including chlorogenic, neochlorogenic, and caffeic acids, have also been identified in water hyssop derived from tissue culture [10]. These findings highlight water hyssop as a promising source of bioactive compounds with potential applications in pharmaceutical and health-related products. Consequently, it serves as a vital upstream raw material for extracting bioactive compounds with significant medical applications.

However, the quantity and composition of plant secondary metabolites are not only determined by genetic factors but also strongly influenced by environmental conditions and cultivation practices. Therefore, understanding the relationship between environmental conditions, particularly water management, and plant growth and bioactive compound accumulation is essential for developing cultivation strategies that can improve the quality, consistency, and concentration of bioactive compounds in water hyssop. In medicinal plants, secondary metabolites represent key bioactive constituents with pharmacological relevance. Their biosynthesis is frequently associated with environmental stress responses [11,12]. Furthermore, the use of elicitation techniques to induce plant stress, such as drought, salinity, thermal, and light stress, can significantly enhance the production of secondary metabolites in medicinal plants. These controlled stress environments often result in higher yields of bioactive compounds compared to plants grown under standard field conditions or in their natural habitats [11,13,14,15,16]. Water deficit is one of the major environmental stresses affecting plant growth and physiological processes. A reduction in soil water availability can alter plant water status, stomatal regulation, gas exchange, and photosynthetic performance, with the severity and duration of water deficit significantly influencing the magnitude of plant responses [17,18]. Water deficit can also disrupt photosynthetic homeostasis and promote the generation of reactive oxygen species (ROS), which have dual roles as both potentially damaging molecules involved in oxidative stress and signaling molecules that activate stress perception and adaptive responses [19,20]. ROS-mediated responses can stimulate antioxidant defense systems and induce metabolic reprogramming, including enhanced synthesis of secondary metabolites such as phenolic compounds. These compounds can function as antioxidants and contribute to cellular protection against oxidative damage under water deficit conditions [21,22]. Therefore, moderate water deficit may act as an eliciting stimulus that induces metabolic acclimation and promotes the accumulation of phenolic compounds. However, plant responses to water deficit are highly dependent on species, stress intensity, and stress duration. When water deficit becomes too severe or prolonged, the capacity for acclimation may be exceeded, resulting in impaired photosynthetic activity, increased oxidative damage, and reductions in biomass production [18,22].

Water hyssop proliferates quickly in wetlands and is naturally found in moist and water-abundant habitats. However, its capacity to acclimate to declining soil water availability and the relationship between water deficit intensity and phenolic accumulation remain insufficiently understood. In particular, the soil moisture level and duration of water restriction required to achieve a balance between physiological acclimation and enhanced accumulation of bioactive compounds have not been clearly established. Identifying an optimal stress level is more informative than simply assessing growth inhibition or physiological damage. Controlled water limitation could potentially be used as a cultivation tool to stimulate secondary metabolite production without compromising plant growth or photosynthetic performance. This approach could provide a basis for precision water management to enhance the accumulation of valuable bioactive compounds in water hyssop. Therefore, this study aimed to characterize the physiological and adaptive responses of water hyssop to different levels and durations of soil water restriction by evaluating changes in morphology, water status, photosynthetic performance, oxidative stress, total phenolic content (TPC), and anatomy. We focused on identifying the relationships among soil water availability, physiological acclimation, and phenolic accumulation. The findings of this study are expected to define a suitable water deficit window that stimulates phenolic accumulation without causing severe physiological damage and to provide a scientific foundation for developing water management strategies to produce water hyssop with enhanced phenolic content and consistent quality.

## 2. Results and Discussion

### 2.1. Effects of Soil Moisture Depletion on Morphological and Physiological Changes

**Figure 1:**
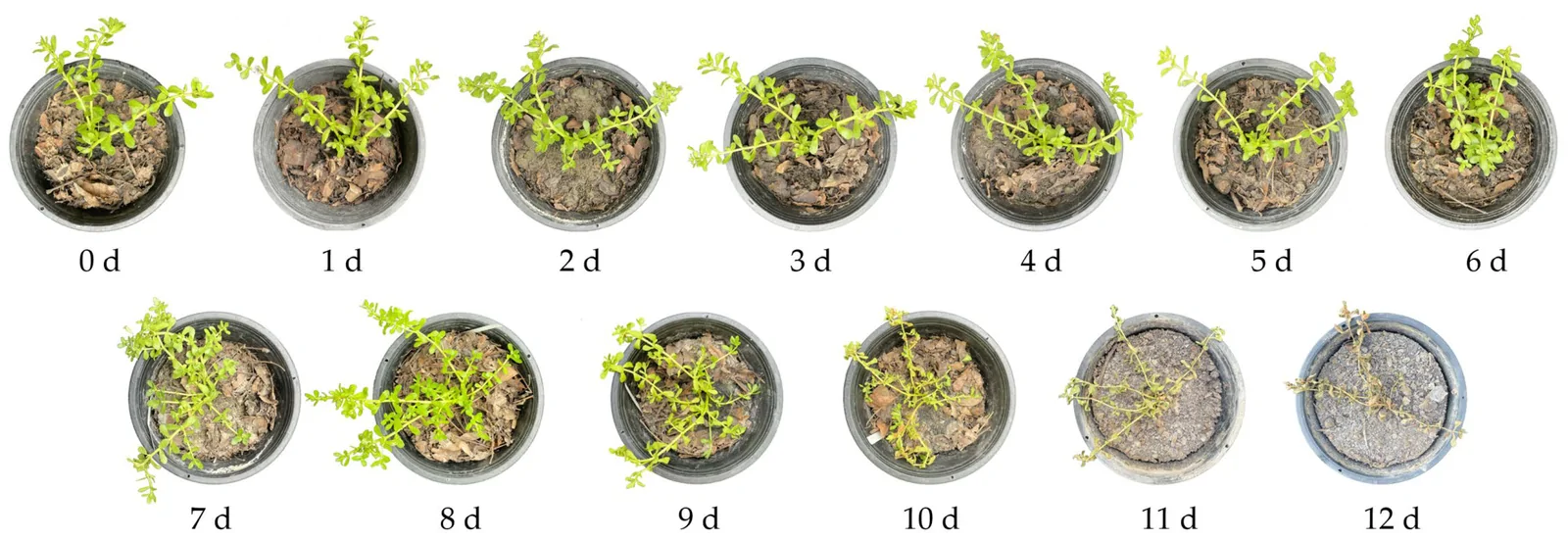
Effect of drought stress on morphological changes in water hyssop after 12 days of culture.

Water hyssop (*Bacopa monnieri*) is an aquatic medicinal plant commonly found along riverbanks and moist habitats, thriving under high soil moisture conditions. Consequently, water deficit imposes severe environmental stress on this species, triggering pronounced morphological and physiological responses. The study revealed that reducing soil moisture by withholding water significantly affected the morphological characteristics of water hyssop (Figure 1). At the onset of the experiment, the water hyssop exhibited optimal health and vigor. The leaves were vibrant green, and the stems remained turgid, upright, and succulent. The soil surface was saturated, characterized by a dark color and a complete absence of surface cracking. In the early stress phase (days 1–6), as the duration of water deprivation increased, the overall appearance of the plant remained relatively similar to its initial state. However, subtle changes began to emerge, such as a slight leaning or loss of verticality in some stems. Simultaneously, the soil began to lose its moisture content, as seen by a noticeable lightening in the soil color and drying of the surface layer. Despite these changes, no significant or clearly visible soil cracking had developed by the end of this period.

In the intermediate stress phase (days 7–9), wilting symptoms became increasingly pronounced. Some leaves began to turn yellow (chlorosis), reflecting a clear physiological response to water deficit, although the stems remained green. During this period, the soil became extremely dry, with a significantly lighter color. Initial surface cracking began to appear in certain areas of the soil.

By the end of the experiment (days 10–12), water hyssop exhibited severe wilting. The leaves transitioned from yellow to brown, becoming brittle and desiccated after 12 days. These symptoms indicate a progression toward permanent wilting and potential plant death. The soil deteriorated into a hard, pale, and arid state, with deep and distinct cracks visible across the entire surface of the pot (Figure 1).

**Figure 2:**
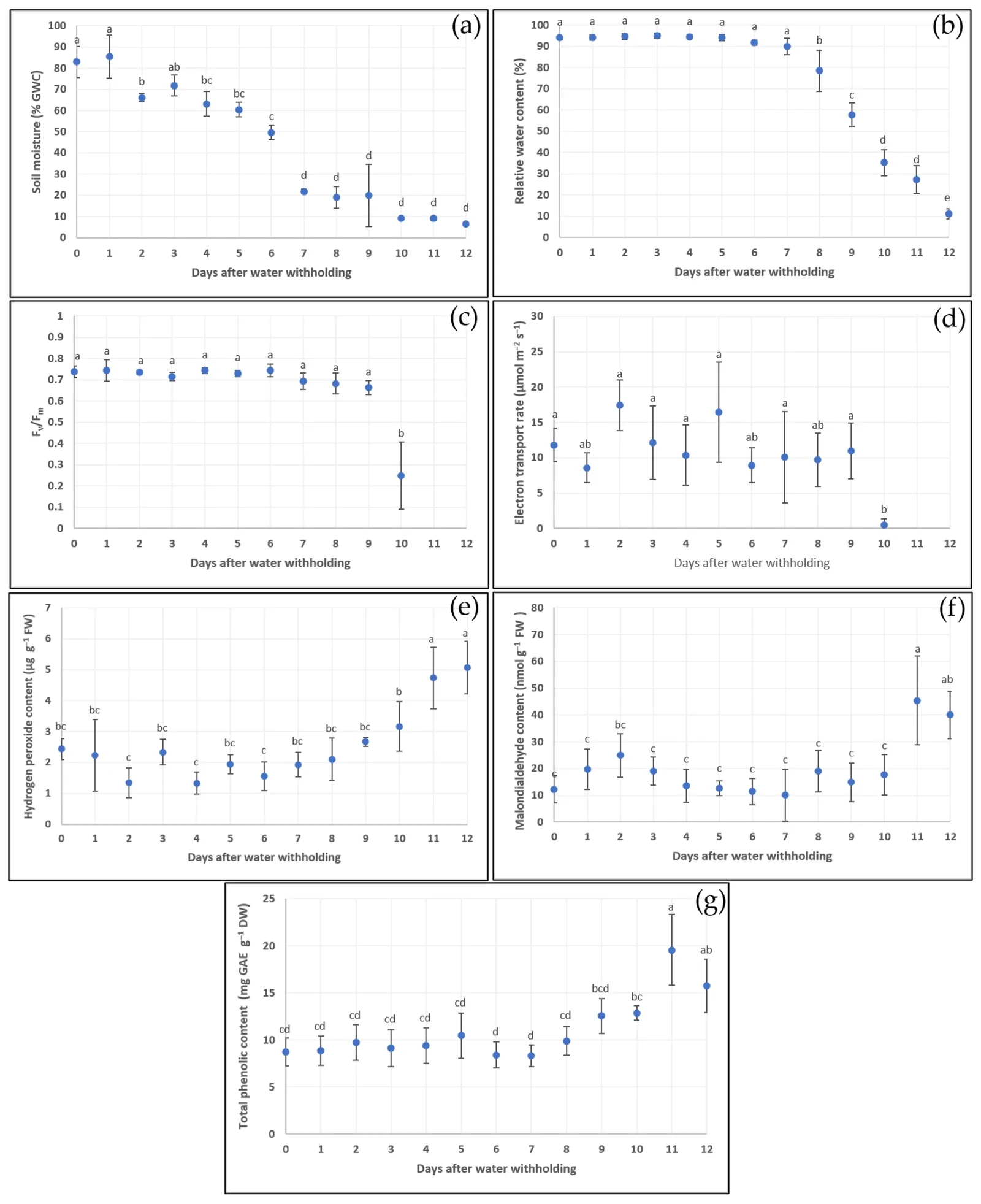
Effects of drought stress on water hyssop. Changes in soil moisture (**a**), relative water content (RWC) (**b**), maximum quantum yield of PSII (F_v_/F_m_) (**c**), electron transport rate (ETR) (**d**), hydrogen peroxide content (H_2_O_2_) (**e**), malondialdehyde (MDA) content (**f**), and total phenolic content (TPC) (**g**) of water hyssop grown under drought stress. Means with the same letter are not significantly different according to Tukey’s HSD test at *p* < 0.05. Data are presented as means ± SD, shown by vertical error bars.

Visual monitoring of the water hyssop and soil surface conditions over a 12-day drought induction period revealed distinct physiological responses as the water deficit intensified. Quantitative analysis revealed that the 12-day water deprivation experiment resulted in a continuous decline in both soil moisture and the plant’s relative water content (RWC). Statistical analysis revealed distinct physiological phases in response to the progressing drought. During the initial phase (days 0–1), soil moisture remained high at 82.89% GWC and 85.36% GWC, respectively. On day 2, a statistically significant drop occurred, with moisture levels falling to 66.12% GWC. On day 7, the moisture content decreased to 21.86% GWC. During days 7–12, following the sharp decline on day 7, soil moisture continued to decrease but showed no further statistical differences during this late stage, reaching a minimum of 6.54% GWC on day 12 (Figure 2a). In contrast to the rapid decline in soil moisture, the RWC of water hyssop remained stable during the first week (Figure 2b). During days 0–7, the RWC stayed consistently high (ranging from 95.09 to 89.88%) with no significant variations despite the significant reduction in soil moisture starting on day 2. During days 8–12, once soil moisture fell below the 21.86% GWC threshold, the RWC began to drop drastically, with percentages of 78.50%, 57.73%, 35.24%, 27.25%, and 11.15%, respectively (Figure 2a,b). The decline in soil moisture was accompanied by reduced photosynthetic performance, as indicated by decreases in the maximum quantum efficiency and yield of photosystem II (F_v_/F_m_) and the electron transport rate (ETR) of water hyssop. From day 1 to day 9 of the drought treatment, Fv/Fm values remained statistically stable, ranging between 0.6626 and 0.7438. This suggests that the structural integrity of photosystem II (PSII) was maintained despite the decline in soil moisture (starting from day 7) and the reduction in the RWC (starting from day 8). However, a marked decline in PSII efficiency was observed on day 10, when the Fv/Fm values decreased significantly to 0.2475 relative to previous days. While soil moisture levels between days 9 and 10 showed no statistically significant difference, RWC declined significantly from 57.73% to 35.24% during this interval. This substantial reduction in tissue hydration was associated with a marked impairment of PSII function. By days 11 and 12, Fv/Fm values became undetectable, reflecting the loss of measurable photochemical activity in the photosynthetic apparatus (Figure 2c). The ETR data followed a similar trend to Fv/Fm. During the initial phase (days 0–9), the ETR values fluctuated slightly but showed no statistically significant differences, with an average of 11.65 µmol m^−2^s^−1^. By day 10, the ETR decreased significantly to 0.45 µmol m^−2^s^−1^, indicating a near-complete inhibition of electron transport. The ETR could no longer be measured on days 11 and 12 (Figure 2d). Similar responses have been reported in plants exposed to drought stress, although the magnitude and direction of changes in chlorophyll fluorescence parameters depend on plant species, stress intensity, and stress duration [23]. Some plants may initially maintain or even increase photosynthetic activity during the early stages of soil drying before experiencing a progressive decline as drought severity increases. Under severe drought, pronounced reductions in chlorophyll fluorescence parameters and ETR indicate impairment of photosynthetic electron transport and PSII function [24]. In the present study, F_v_/F_m_ values of water hyssop under well-watered conditions were already below the commonly reported range of 0.80–0.83 for non-stressed terrestrial plants [25]. Furthermore, none of the soil moisture treatments resulted in F_v_/F_m_ values exceeding 0.75 (Figure 2c). Therefore, the commonly cited F_v_/F_m_ threshold should not be directly applied as an absolute criterion for defining drought stress in water hyssop. Instead, changes relative to the species-specific baseline under well-watered conditions provide a more appropriate indicator of PSII responses to drought stress. A pronounced decline in both F_v_/F_m_ values and the ETR occurred when soil moisture decreased to 9.10% GWC, whereas these parameters remained relatively stable at higher soil moisture levels (Figure 2a,c,d). This finding suggests that 9.10% GWC may represent a critical threshold at which photosynthetic performance becomes substantially impaired in water hyssop. In contrast, plant water status, as indicated by the RWC, began to decline at a considerably higher soil moisture level of 21.86% GWC and continued to decrease progressively with further soil drying.

Continuous water deprivation was associated with changes in varying levels of physiological stress in water hyssop, as indicated by the accumulation of hydrogen peroxide (H_2_O_2_) and malondialdehyde (MDA) (Figure 2e,f). During the initial phase of the drought treatment (days 0–9), H_2_O_2_ levels showed no statistically significant differences, with an average concentration of 1.99 µg g^−1^ fresh weight. On day 10, a rising trend was observed as H_2_O_2_ levels increased to 3.17 µg g^−1^ fresh weight. On days 11 and 12, H_2_O_2_ increased substantially to 4.74 and 5.07 µg g^−1^ fresh weight, respectively. These values represent a statistically significant increase compared to the previous days, signaling severe oxidative stress (Figure 2e). The accumulation of MDA followed a similar pattern to that of H_2_O_2_. During days 0–10, there were no significant differences in MDA levels, which averaged 15.96 nmol g^−1^ fresh weight. On days 11 and 12, MDA concentrations reached peak levels of 45.37 and 39.98 nmol g^−1^ fresh weight, respectively, which are significantly higher than those recorded during the first nine days of the experiment (Figure 2f). These results indicate that prolonged water deprivation was associated with elevated H_2_O_2_ levels in water hyssop tissues. This rise in ROS coincided with the significant increase in MDA levels on days 11 and 12. Collectively, these observations indicate that extended water deprivation is associated with heightened oxidative stress markers during the final desiccation stage. Hydrogen peroxide (H_2_O_2_) is a prominent reactive oxygen species (ROS) recognized in the literature as both a potential signaling molecule and an indicator of oxidative stress when elevated. Meanwhile, malondialdehyde (MDA) is a secondary product of lipid peroxidation widely utilized as a marker for oxidative membrane damage under abiotic stress [26]. It should be noted, however, that because these parameters were expressed on a fresh weight and concentration basis, the apparent increases during severe stress (days 11–12) may be partially amplified by tissue dehydration. Nevertheless, the concurrent elevation of both markers is consistent with heightened oxidative stress during late-stage water deficit.

During the initial period of water deprivation (days 0–8), the total phenolic content (TPC) remained statistically stable, with an average concentration of 9.21 mg GAE g^−1^ DW. On day 9, a rising trend emerged as the TPC increased to 12.56 mg GAE g^−1^ DW. On day 11, the TPC reached its peak concentration of 19.57 mg GAE g^−1^ DW when soil moisture dropped to 9.06% GWC. On day 12, when soil moisture dropped to a minimum of 6.54% GWC, TPC levels plateaued and showed no statistically significant difference from day 11 levels (Figure 2g). These results indicate that reducing soil moisture is closely associated with an increased accumulation of phenolic compounds. The peak TPC value observed at 9.06% GWC corresponds to maximal phenolic accumulation during progressive soil drying. However, as soil moisture fell further to approximately 7% GWC, TPC levels remained statistically comparable without significant increases, confirming that phenolic accumulation plateaus under extreme water deficit. Furthermore, visual symptoms of drought stress included wilting and a distinct color transition from green to yellow and brown. At 9.06% GWC, the leaves exhibited severe wilting and turned brown. At this stage, the plant reached its maximum observed total phenolic content (TPC). These findings indicate that drought stress promotes secondary metabolite accumulation in water hyssop. As this species naturally thrives in riparian and wetland habitats, water deprivation led to distinct morphological and physiological alterations.

The relationship between RWC and photosynthetic parameters indicates that PSII photochemistry and electron transport were maintained despite a substantial decline in tissue hydration. However, once soil moisture declined to 9.10% GWC (days 10), both F_v_/F_m_ values and ETR decreased markedly, reflecting a pronounced reduction in photosynthetic efficiency. Under extreme water deficit (6.54% GWC), TPC levels remained statistically comparable to those observed at 9.06% GWC, coinciding with progressive tissue dehydration and visible desiccation. This pattern demonstrates that phenolic accumulation plateaus rather than continuing to increase during late-stage drought. Overall, these findings highlight a concurrent sequence of physiological and biochemical changes in water hyssop—characterized by tissue dehydration, reduced photosynthetic performance, elevated oxidative stress markers, and increased TPC. Phenolic compounds are non-enzymatic metabolites commonly associated with ROS scavenging capacity and cellular redox status under water limitation [27]. Consistently, increases in total phenolic content (TPC) during drought stress have been documented across various plant species [28,29]. However, such biochemical responses vary depending on phenolic composition, tissue type, stress duration and severity, as well as plant species. Consequently, an elevation in TPC is not a universal marker of drought tolerance.

**Figure 3:**
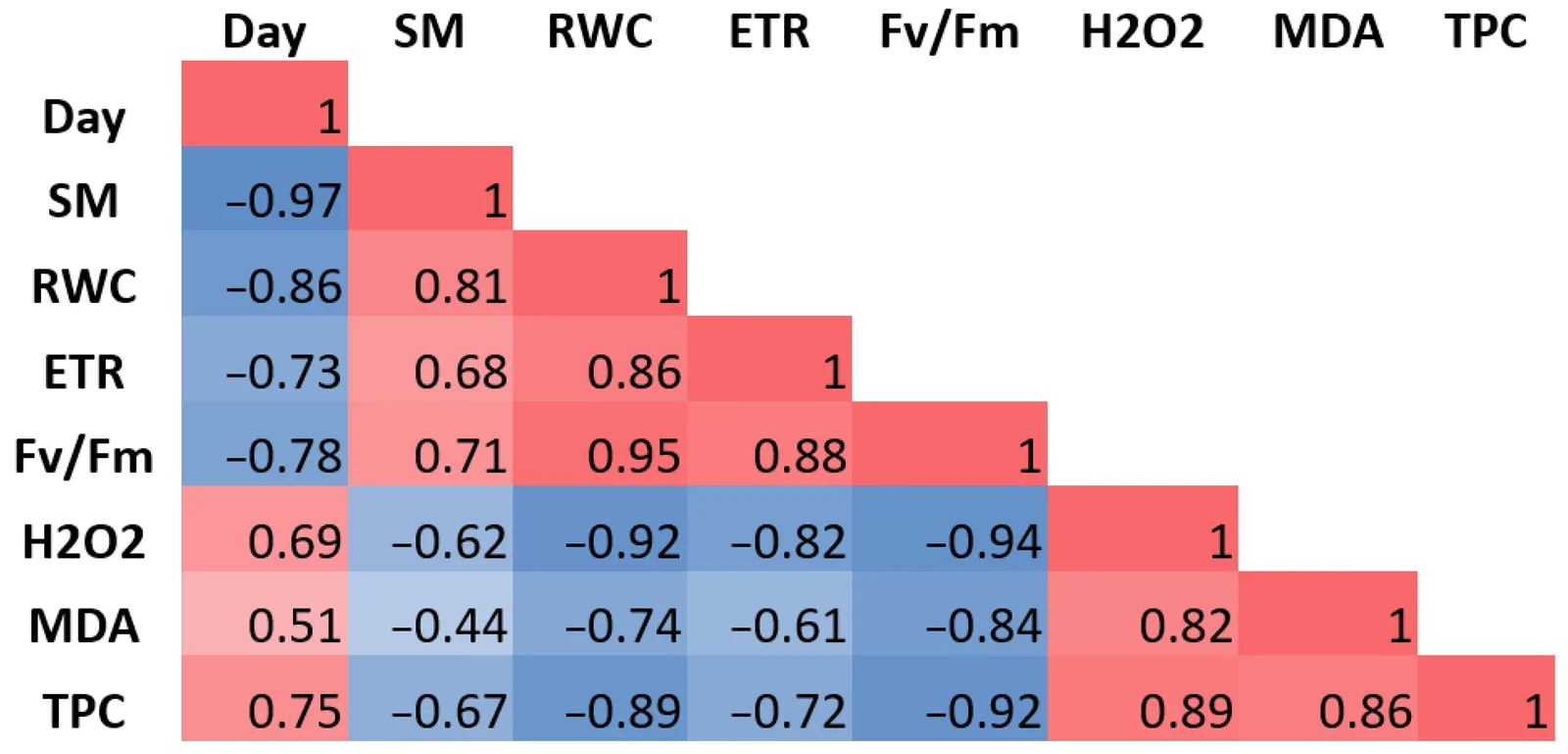
Correlation analysis of physiological parameters of water hyssop under drought stress. Values are correlation coefficients (r); red and blue cells indicate positive and negative correlations, respectively, and color intensity reflects the strength of the correlation. Day, days after the onset of drought stress; SM, soil moisture; RWC, relative water content; ETR, electron transport rate; F_v_/F_m_, maximum quantum yield of PSII; H_2_O_2_, hydrogen peroxide; MDA, malondialdehyde; TPC, total phenolic content.

In the present study, the total phenolic content (TPC) positively correlated with H_2_O_2_ (r = 0.89) and MDA (r = 0.86), indicating that increasing oxidative stress was associated with enhanced accumulation of phenolic compounds (Figure 3). The increase in TPC under moderate-to-severe drought is consistent with the general observation that plants often accumulate antioxidant metabolites during environmental stress. However, the finding that TPC levels plateaued rather than increasing further under extreme drought suggests that phenolic accumulation is constrained when the stress exceeds the plant’s physiological tolerance.

Among the physiological parameters evaluated, the relative water content (RWC) was found to be a sensitive indicator of decreasing soil moisture in water hyssop. The decline in internal water content coincided with a significant reduction in photosynthetic efficiency and an accumulation of oxidative stress markers. These associations were supported by strong negative correlations between Fv/Fm and both H_2_O_2_ (r = −0.94) and MDA (r = −0.84). Similarly, the electron transport rate (ETR) exhibited a high negative correlation with H_2_O_2_ (r = −0.82) and a moderate negative correlation with MDA levels (r = −0.61) (Figure 3).

### 2.2. Effects of Prolonged Drought Stress

#### 2.2.1. Morphological Response

**Figure 4:**
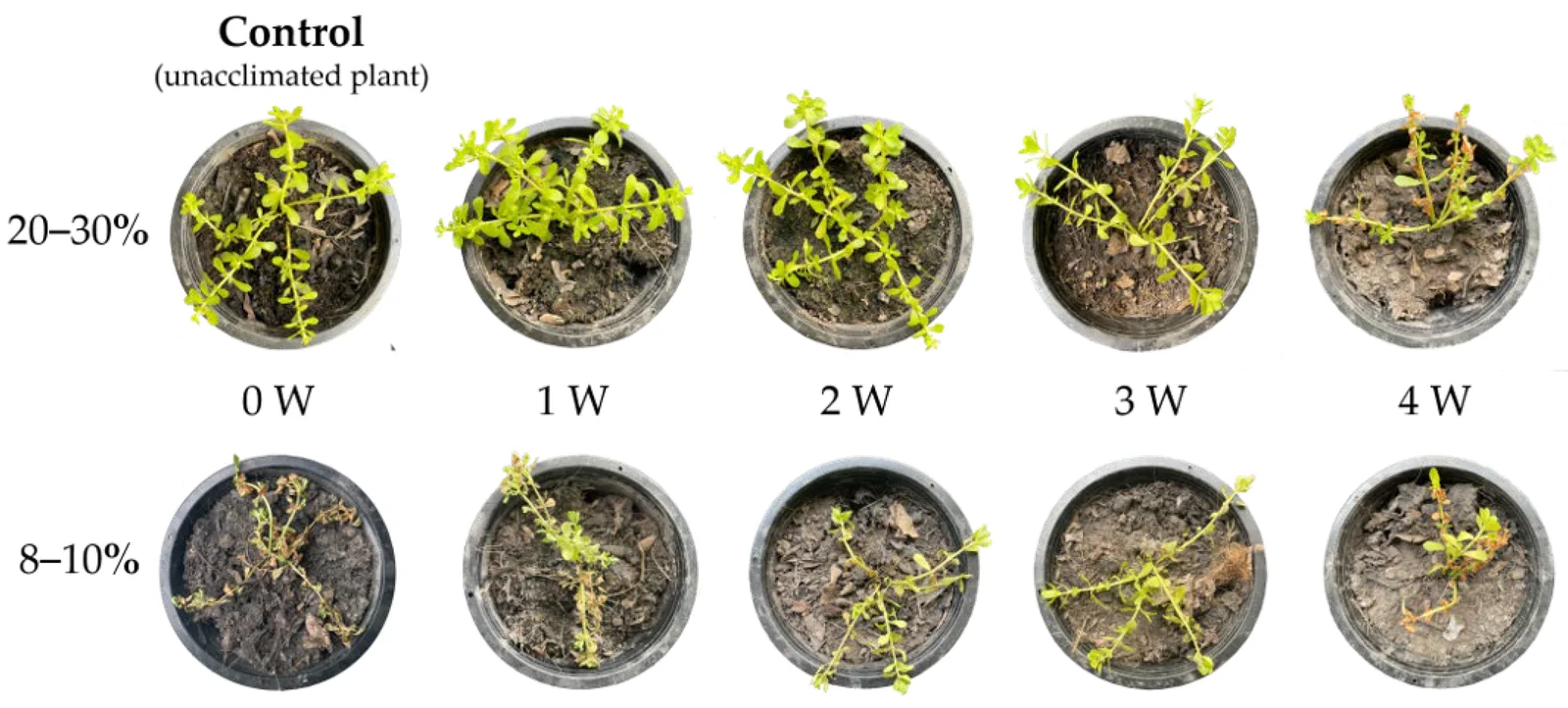
Morphological response of water hyssop maintained at 20–30% GWC and 8–10% GWC for 0, 1, 2, 3, and 4 weeks. Measurements were recorded weekly over a 4-week period.

**Figure 5:**
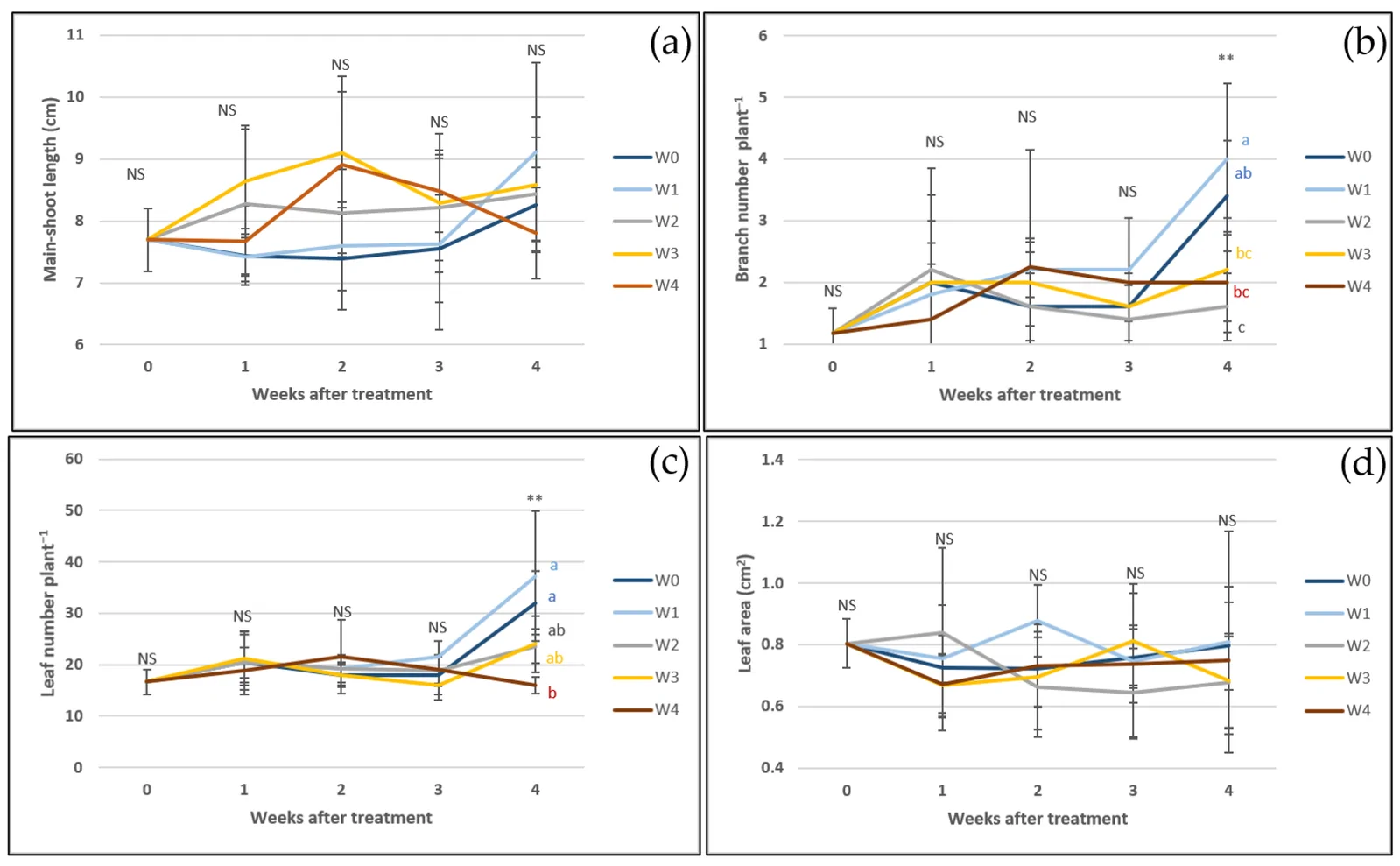
Morphological responses of water hyssop, including main shoot length (**a**), branch number (**b**), leaf number (**c**), and leaf area (**d**) under continuous soil moisture conditions maintained at 20–30% GWC for 0 (W0), 1 (W1), 2 (W2), 3 (W3), and 4 (W4) weeks. Measurements were recorded weekly over a 4-week period. NS indicates no statistically significant difference, whereas ** indicates a statistically significant difference at the 99% confidence level, as determined by Tukey’s multiple comparison test. Different letters indicate statistically significant differences among treatments.

Water hyssop plants were maintained at 20–30% GWC for 0–4 weeks and subsequently exposed to 8–10% GWC to evaluate drought acclimation and phenolic accumulation. During sustained exposure to 20–30% GWC, plants maintained for 0–2 weeks remained green and vigorous, whereas those exposed for 3 weeks exhibited lighter green, soft, and wilted leaves, and those exposed for 4 weeks showed extensive leaf browning, although newly formed branches remained green. Stem appearance remained statistically and visually comparable across treatments (Figure 4 and Figure 5). Following subsequent exposure to 8–10% GWC, unacclimated plants (0 weeks) exhibited the most severe wilting and desiccation, whereas plants previously exposed to 20–30% GWC for 1–4 weeks retained higher proportions of green tissue, suggesting that prior exposure to moderate water deficit was associated with enhanced tissue preservation under severe stress. Prolonged exposure to 20–30% GWC was associated with significantly lower branch and leaf numbers, particularly after 2–4 weeks, whereas main shoot length and leaf size showed no statistically significant differences across treatments (Figure 5). Reductions in shoot and leaf numbers correspond to documented structural adjustments associated with a diminished transpiring surface area [30,31,32,33]. Overall, the retention of main shoot length and leaf size alongside lower organ counts indicates that water hyssop exhibits selective structural variations under prolonged low soil moisture, maintaining primary shoot extension despite lower rates of new branch and leaf formation.

#### 2.2.2. Physiological Responses

**Figure 6:**
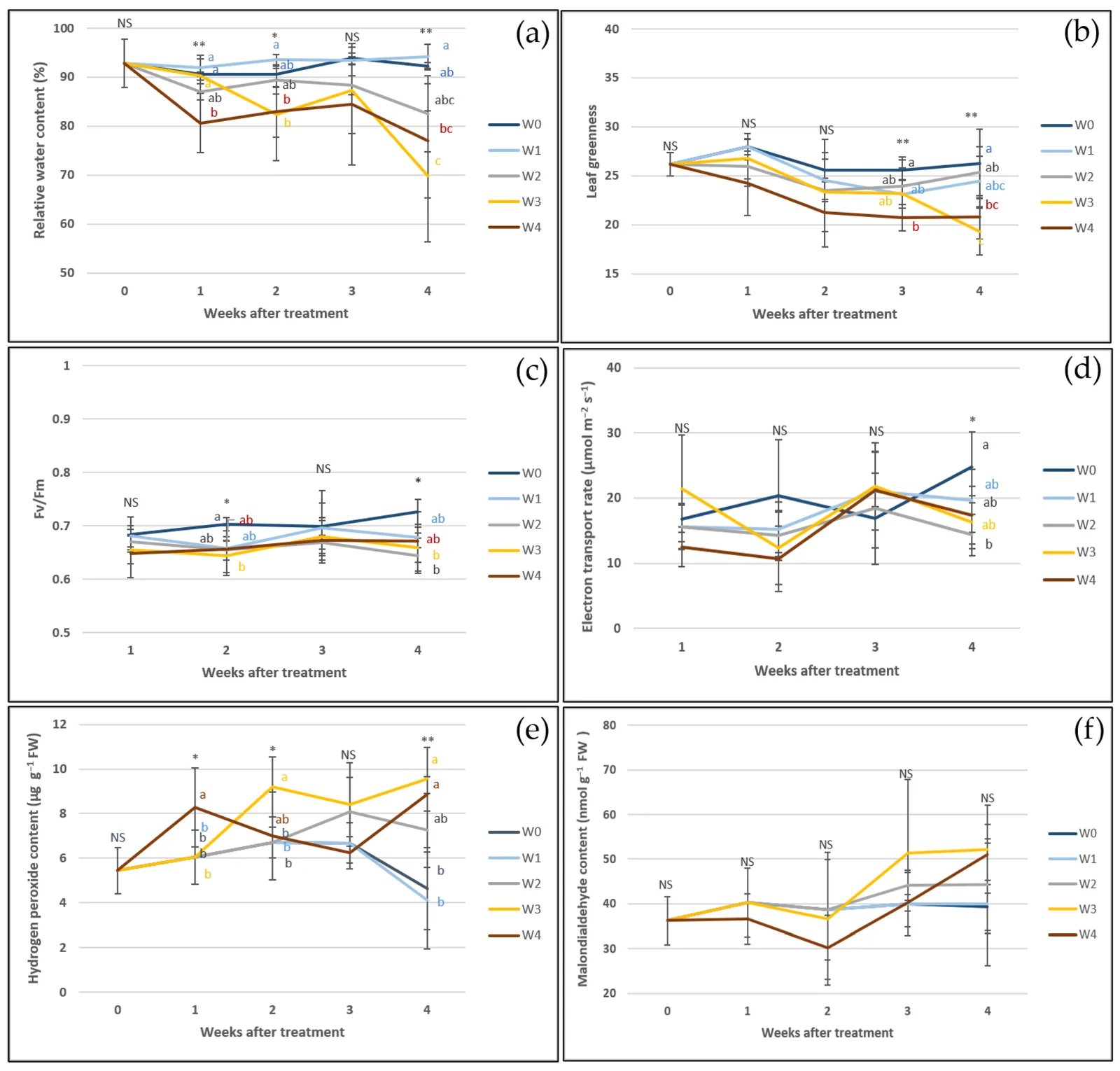
Physiological responses of water hyssop, including relative water content (RWC) (**a**), leaf greenness (**b**), maximum quantum efficiency of photosystem II (Fv/Fm) (**c**), electron transport rate (ETR) (**d**), hydrogen peroxide (H_2_O_2_) (**e**), and malondialdehyde (**f**), under continuous soil moisture conditions maintained at 20–30% GWC for 0 (W0), 1 (W1), 2 (W2), 3 (W3), and 4 (W4) weeks. Measurements were recorded weekly over a 4-week period. NS indicates no statistically significant difference, whereas * and ** indicate statistically significant differences at the 95% and 99% confidence levels, respectively, as determined by Tukey’s multiple comparison test. Different letters indicate statistically significant differences among treatments.

Water hyssop exhibited time-dependent physiological responses during prolonged moderate soil moisture restriction (20–30% GWC). Relative water content (RWC) and leaf greenness remained statistically stable under moderate soil moisture deficit for 1–3 weeks but decreased after 4 weeks, coinciding with progressive tissue water loss under extended treatment (Figure 6a,b). Throughout the 4-week period of continuous water restriction, F_v_/F_m_ values and the electron transport rate (ETR) remained continuously stable and statistically comparable to control levels, showing no significant temporal decline or subsequent rebound across time points (Figure 6c,d). This stability is consistent with literature reporting maintained PSII photochemistry during moderate water deficit [34,35]. Prolonged stress was also associated with increased H_2_O_2_ accumulation after 3–4 weeks (Figure 6e), whereas malondialdehyde (MDA) levels remained statistically unchanged (Figure 6f). Collectively, water hyssop exhibits effective physiological adaptations and tolerance to prolonged moderate water deficit. Specifically, this plant maintains the stability of its photosystem II (PSII) photochemistry over the full 4-week period, even though extended water restriction (>2 weeks) leads to tissue water loss, decreased leaf greenness, and increased H_2_O_2_ accumulation.

#### 2.2.3. Biomass Production and Phenolic Accumulation

**Figure 7:**
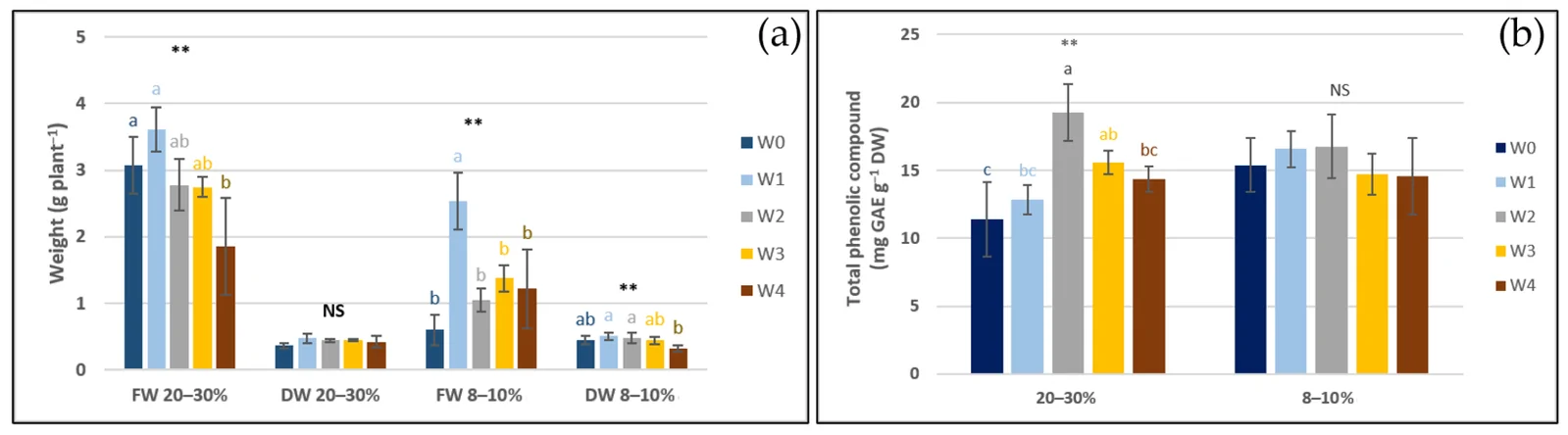
Yield of water hyssop plants, including fresh (FW) and dry weights (DW) (**a**) and total phenolic content (TPC) (**b**) under continuous soil moisture conditions maintained at 20–30% GWC for 0 (W0), 1 (W1), 2 (W2), 3 (W3), and 4 (W4) weeks. Measurements were performed at 4 weeks after the initiation of drought stress and again when soil moisture reached 8–10% GWC. NS indicates no statistically significant difference, whereas ** indicates a statistically significant difference at the 99% confidence level, as determined by Tukey’s multiple comparison test. Different letters indicate statistically significant differences among treatments.

Maintaining soil moisture at 20–30% GWC for 1–3 weeks was associated with no significant alterations in the fresh or dry biomass of water hyssop relative to the well-watered control. Conversely, extending water limitation to 4 weeks resulted in a significantly lower fresh weight compared to the control group (Figure 7a). Notably, after soil moisture was reduced to 8–10% GWC, plants pre-exposed to 1 week of water restriction exhibited a significantly higher fresh weight than both control plants and those in other treatment groups. Meanwhile, the dry weight of plants restricted for 1–4 weeks remained statistically comparable to control levels. These results show that early exposure to moderate stress prepares plants to better survive later severe droughts [36,37,38,39]. Regarding secondary metabolites, water restriction for 2–3 weeks was associated with significantly higher total phenolic content (TPC), whereas TPC levels after 1 and 4 weeks were statistically comparable to control levels, indicating that phenolic accumulation varies with both stress intensity and duration rather than exhibiting a continuous linear increase over time. Moderate water deficit is frequently reported in the literature to coincide with elevated secondary metabolite accumulation and antioxidant responses [22,40]. However, the absence of further TPC accumulation after 4 weeks in the present study indicates that phenolic levels reach a plateau rather than continuing to rise under sustained stress. During the moderate water-deficit stage (20–30% GWC), TPC reached a peak concentration of 19.26 mg GAE g^−1^ DW in the W2 treatment (Figure 7b). However, following subsequent soil drying to the severe water-deficit stage (8–10% GWC), TPC levels showed no significant increases and remained statistically comparable across treatments. Notably, plants previously exposed to moderate water limitation (W1–W4; 20–30% GWC) reached the severe drought threshold (8–10% GWC) within 4 days of complete water withholding, compared to 11 days for well-watered controls (W0). This accelerated drying coincided with retained biomass and tissue hydration during early stress. Specifically, W1 plants maintained significantly higher fresh weight under severe drought than controls. Total phenolic content (TPC) exhibited stage-specific accumulation, peaking at W2 under moderate water restriction (20–30% GWC). However, upon rapid progression to severe drought (8–10% GWC), TPC levels remained comparable to the W2 peak without further accumulation. Consequently, the physiological and biochemical responses recorded at 8–10% GWC reflect the integrated outcome of prior stress exposure duration, the preservation of plant hydration status, and the accelerated rate of soil drying. Overall, these findings suggest that moderate restriction at 20–30% GWC for 2 weeks provides an optimal window for TPC accumulation without a biomass penalty, whereas the rapid onset of severe drought yields no additional biochemical benefit and coincides with lower fresh biomass.

#### 2.2.4. Anatomical Acclimation

**Figure 8:**
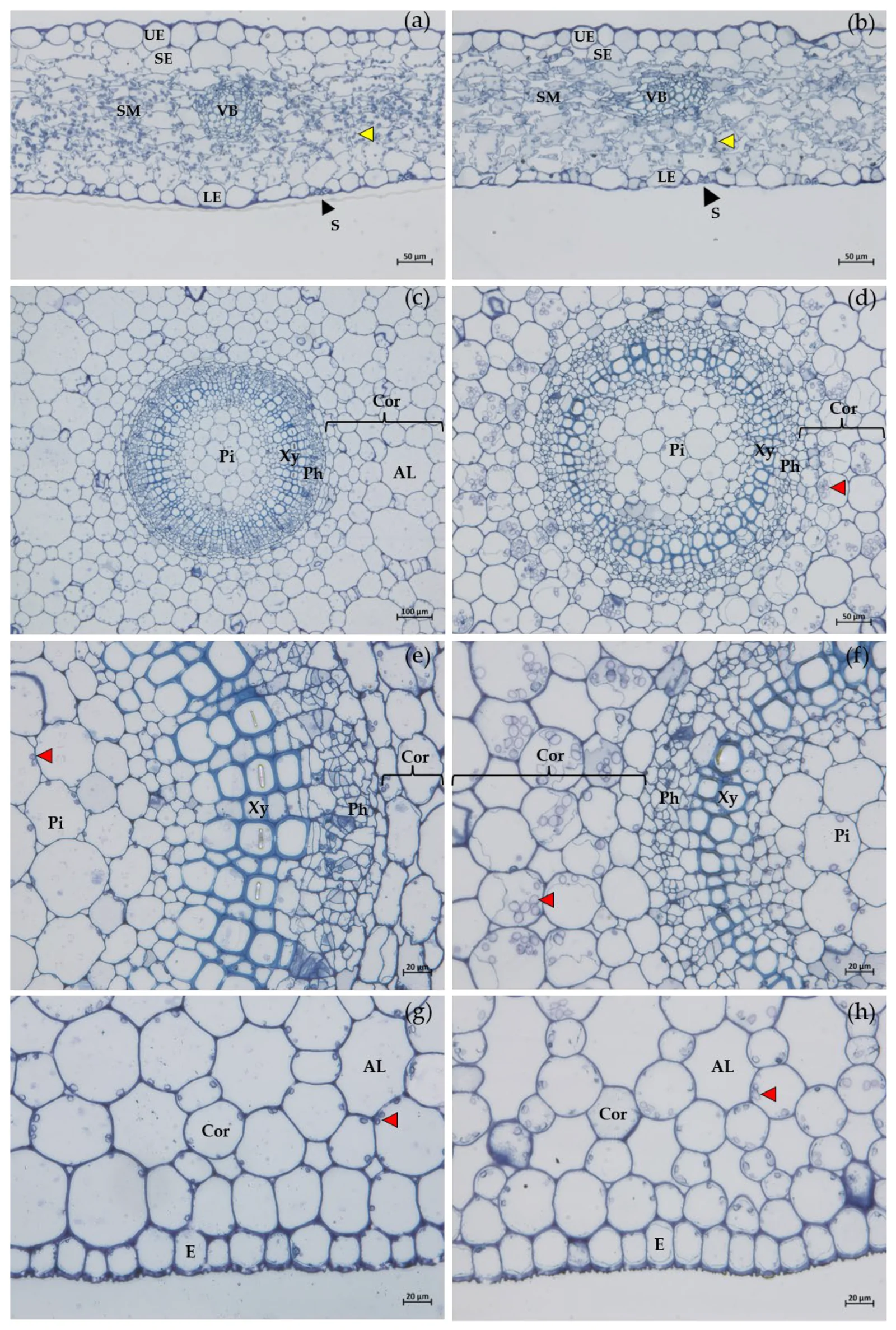
Anatomical changes in the leaves (**a**,**b**) and stems (**c**–**h**) of water hyssop maintained at 20–30% GWC for 0 (**a**,**c**,**e**,**g**) (W0) and 4 weeks (**b**,**d**,**f**,**h**) (W4). AL, air lacunae; Cor, cortex; E, epidermis; LE, lower epidermis; Pi, pith; Ph, phloem; S, stoma; SE, sub-epidermis; SM, spongy mesophyll; Xy, xylem; UE, upper epidermis; VB, vascular bundle; yellow arrow, chloroplast; red arrow, starch grain.

Although water hyssop sustained growth under moderate water limitation (20–30% GWC) for up to four weeks, prolonged restriction triggered pronounced physiological and anatomical adjustments in both the leaves and stems (Figure 8), including a reduction in the area of leaf epidermal and sub-epidermal cells. Additionally, structural modifications were observed in the vascular tissues and mesophyll layer, characterized by cellular flattening and a loss of regular conformation relative to the control group (Figure 8a,b). An increase in mesophyll intercellular spaces and a marked reduction in chloroplast abundance were also observed, consistent with the decline in relative water content (RWC) and leaf greenness, together with the tendency toward lower F_v_/F_m_ values and ETR, indicating impairment of plant water status and photosynthetic performance under prolonged water limitation (Figure 8a,b). Reduced cellular turgor under limited water availability may restrict cell expansion and contribute to the observed anatomical modifications, whereas changes in vascular bundle and xylem structure may represent adjustments of the water-conducting system to maintain leaf water balance [41,42]. The increased mesophyll intercellular spaces may further reflect structural acclimation to water limitation and potentially influence CO_2_ diffusion and photosynthetic performance [43]. After 4 weeks of water restriction, leaf anatomical observations revealed reduced chloroplast abundance (Figure 8b). This decline correlated with decreased photosynthetic performance and increased H_2_O_2_ concentration. Nevertheless, the MDA content remained statistically unchanged from the controls, suggesting that oxidative stress occurred without inducing severe membrane lipid peroxidation [43,44]. In stems, water limitation was associated with reduced intercellular spaces in the cortex, altered shape of cortical cells adjacent to vascular bundles, enhanced starch grain abundance around vascular bundles, and a marked decrease in xylem vessel size (Figure 8c–h). The reduced intercellular spaces and altered cortical cell morphology may be associated with restricted cell expansion under reduced water availability. Meanwhile, the increased starch accumulation likely reflects changes in carbohydrate storage or utilization as plant growth becomes constrained. Furthermore, the reduction in xylem vessel size observed in both leaves and stems may represent an anatomical adjustment to declining water availability [45,46,47]. Collectively, the coordinated changes in leaf mesophyll structure, vascular tissues, chloroplast abundance, and starch storage demonstrate that water hyssop employs multiple levels of anatomical and physiological acclimation to sustained drought. These adjustments help preserve tissue hydration and physiological function despite severe water limitation.

## 3. Materials and Methods

### 3.1. Plant Material and Experimental Set-Up

Healthy mother plants of *Bacopa monnieri* were collected from their natural habitat at the Faculty of Fisheries, Kasetsart University, Thailand. The voucher specimen of *B. monnieri* (voucher no. 084795) was deposited in the Bangkok herbarium, Department of Agriculture, Thailand. Shoot cuttings of water hyssop (*B. monnieri*) approximately 10 cm in length were propagated in peat moss for 2 weeks to facilitate root development. The rooted cuttings were subsequently transplanted into pots with a top diameter of 7 inches containing well-moistened soil (the soil mixture exhibited a high organic matter (OM) content of 8.5–10.5%, a bulk density of approximately 0.8 g cm^−3^, and a pH of 6.5). To ensure high soil moisture, pots were placed in trays inside the greenhouse for two weeks. The experiment was conducted in May under an average daytime photosynthetic photon flux density (PPFD) of 602.83 µmol m^−2^s^−1^, as measured by a Li-COR Spectrometer (LI-COR, Inc., Lincoln, NE, USA). During this period, the average daytime and nighttime temperatures were 35–38 °C and 25–27 °C, respectively, with an average RH of 70–80%. Based on these values, the calculated daytime and nighttime vapor pressure deficits (VPD) were 1.57 kPa and 0.84 kPa, respectively.

### 3.2. Drought Stress Treatments and Experimental Design

Two drought stress experiments were conducted. The first experiment was performed to determine the soil moisture threshold that induced water stress and promoted the accumulation of bioactive compounds, particularly phenolic compounds. The second experiment was conducted to investigate plant adaptation to prolonged drought stress. The soil moisture was determined using the gravimetric method. Gravimetric water content (GWC, %) was determined based on the dry mass of the soil substrate. Prior to experimental setup, the tare weight of empty pots was recorded, and the initial dry mass of the soil substrate was measured prior to planting. The dry mass of the soil substrate (W_dry soil_) was determined following oven-drying at 70 °C until a constant weight was achieved. GWC was calculated using the following Equation (1) [48]:

Gravimetric Water Content (% GWC) = [(W_moist soil_ − W_dry soil_)/W_dry soil_] × 100 (1)

where W_moist soil_ is the mass of moist soil and W_dry soil_ is the mass of soil dried at 70 °C to a constant weight.

The experiments were arranged in a completely randomized design (CRD). Each experimental treatment and concurrent well-watered control group comprised independent pots containing a single plant per pot, which served as the primary biological experimental unit (*N* = 130 total pots for Experiment 1; *N* = 50 total pots for Experiment 2). At each sampling time point, ten independent biological replicates (*n* = 10 pots) per treatment and concurrent control group were randomly selected for non-destructive physiological measurements, followed immediately by destructive harvesting for biomass, morphological, anatomical, and biochemical (total phenolic content, hydrogen peroxide, and malondialdehyde) analyses. For leaf morphological and physiological assays, 10 leaves were measured per plant as technical sub-samples and averaged to yield a single representative mean per biological replicate (*n* = 1 value per pot) prior to statistical analysis.

In experiment 1, during plantlet establishment, pots were kept in water-filled trays to provide continuous sub-irrigation for optimal growth. Bottom-irrigation was applied exclusively during this preparation period. To initiate drought stress, all pots were removed from the trays, allowing soil moisture to gradually decrease under greenhouse conditions. Plant samples were randomly collected at daily intervals following the onset of water deficit until severe wilting and complete browning of both leaves and stems occurred on day 12 (Figure 1). Drought conditions were induced by withholding irrigation. The soil moisture level associated with the onset of visible wilting was subsequently used to define the drought stress threshold for the subsequent experiment.

**Figure 9:**
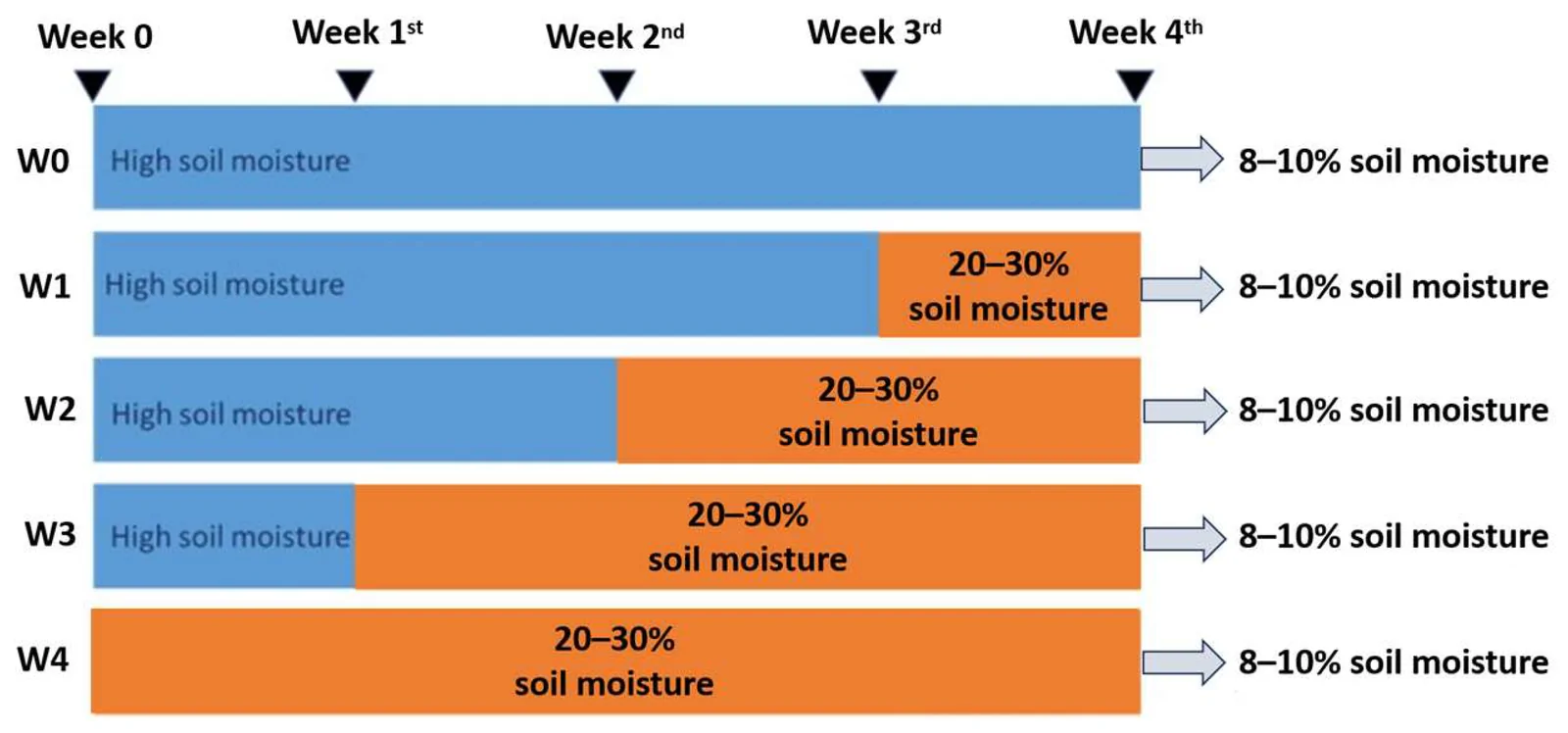
Experimental design of water restriction treatments in water hyssop. W0: maintained at high soil moisture for 4 weeks (unacclimated control); W1–W4: maintained at moderate water deficit (20–30% GWC) for 1, 2, 3, and 4 weeks, respectively. All treatments concluded with a soil moisture reduction to 8–10% GWC prior to harvest. Blue bars indicate high soil moisture, orange bars indicate moderate water deficit (20–30% GWC), and grey arrows indicate the final reduction of soil moisture to 8–10% GWC.

In experiment 2, based on the results of Experiment 1, water hyssop plants began to exhibit distinct physiological responses to water deficit when soil moisture declined to approximately 21.86% GWC, whereas maximum total phenolic content was observed at 9.05% GWC. Therefore, experiment 2 was designed to investigate the effects of drought acclimation duration on plant responses to subsequent water deficit. The experiment consisted of two sequential phases. In the first phase, plants were maintained under moderate water-deficit conditions (20–30% GWC) for 0, 1, 2, 3, or 4 weeks to promote physiological acclimation. To monitor soil moisture non-destructively without disturbing the plant root systems, a custom Smart IoT moisture monitoring and control system was utilized. This system, co-developed by the Kasetsart Agricultural and Agro-Industrial Product Improvement Institute (Kasetsart University) and King Mongkut’s University of Technology Thonburi, employed real-time telemetry to apply targeted irrigation, thereby maintaining soil moisture strictly within the 20–30% GWC range during moderate drought treatments. In the second phase, irrigation was withheld to allow soil moisture to further decline to a target of 8–10% GWC prior to harvest to stimulate phenolic accumulation. The transition to this severe water-deficit level took 4 days for the previously acclimated plants (W1–W4), while the unacclimated plants (W0) took 11 days. Sampling was standardized to the physiological target soil moisture threshold of 8–10% GWC, representing the critical stress point for water hyssop, rather than to fixed time intervals. This ensured that biochemical and physiological parameters were evaluated at equivalent plant-available soil moisture levels across all treatments. This experimental approach was intended to determine whether different durations of prior exposure to moderate water deficit could influence plant physiological performance and phenolic accumulation under subsequent, more severe water-deficit conditions. (Figure 9).

### 3.3. Morphological Study and Biomass Measurements

The morphological characteristics of water hyssop were evaluated using random samples with 10 replicates per treatment. We examined structural features under a stereomicroscope with a camera (Olympus SZ 40; Olympus Corporation, Tokyo, Japan) and measured shoot length, number of branches, leaf number, and leaf area. Daily tracking was supported by representative images. Fresh and dry weights were determined when soil moisture reached 8–10% GWC after 4 weeks of drought treatment. This target level was attained on day 4 for W1–W4 and day 11 for unacclimated plants (W0).

### 3.4. Assessment of Physiological Parameters

Physiological parameters were measured daily during the first experiment at the initiation of drought stress until the plants exhibited visible wilting symptoms. In the second experiment, the same parameters were measured at 0, 1, 2, 3, and 4 weeks after the initiation of drought stress. The measured parameters included leaf greenness, photosynthetic efficiency, and soil moisture, relative water (RWC), hydrogen peroxide (H_2_O_2_), and malondialdehyde (MDA) contents. Leaf greenness was quantified using a portable chlorophyll meter (SPAD-502; Minolta Corp., Kyoto, Japan) on the first to third fully expanded leaves of each plant. Photosynthetic performance and RWC were assessed using the same leaves. Photosynthetic performance was measured using a pulse-amplitude-modulated fluorometer (PAM-2500; Walz, Effeltrich, Germany). The maximum quantum efficiency of photosystem II (Fv/Fm) and the electron transport rate (ETR) were recorded. The RWC was determined according to Equation (2) [49]:

Leaf RWC (%) = [(FW − DW)/(TW − DW)] × 100(2)

where FW is the fresh weight, TW is the turgid weight, and DW is the dry weight. The FW was recorded immediately after leaf excision. The samples were then immersed in distilled water for 24 h to determine TW and subsequently oven-dried at 70 °C until a constant weight was achieved to obtain DW.

Hydrogen peroxide (H_2_O_2_) content was determined following the method of [50] with modifications. Briefly, 0.1 g of fresh tissue, the first to tenth fully expanded leaves, counting from the shoot apex, along with the stem region bearing these leaves, were collected for analysis, was homogenized in 1.5 mL of 0.1% (*w*/*v*) trichloroacetic acid (TCA) and centrifuged at 12,000× *g* for 15 min at 4 °C. A 0.5 mL aliquot of the supernatant was mixed with 0.5 mL of potassium phosphate buffer (pH 7.0) and 1 mL of potassium iodide solution. The reaction mixture was incubated at room temperature for 20 min. Absorbance was measured at 390 nm using a microplate reader. H_2_O_2_ concentration was calculated based on a standard calibration curve. The H_2_O_2_ concentration was quantified using an equation derived from a standard calibration curve of H_2_O_2_ at concentrations of 0, 50, 100, 150, and 200 μM.

Malondialdehyde (MDA) assay was performed, and lipid peroxidation was estimated by determining the MDA content [51]. The sample (0.1 g) was ground in liquid nitrogen and homogenized in 2 mL of 0.1% (*w*/*v*) TCA. The homogenate was centrifuged at 12,000× *g* for 15 min at 4 °C. The supernatant was mixed with 1 mL of 0.5% (*w*/*v*) thiobarbituric acid (TBA) solution and incubated at 98 °C for 30 min. The reaction was terminated by cooling to room temperature. Absorbance was measured at 532 and 600 nm, and the MDA concentration was calculated using Equation (3):

MDA (µmol g^−1^ FW) = [(A_532_ − A_600_)/155] × (V_TCA_/V_sample_) × (V_sample_ + V_TBA_)/W (3)

where V_TCA_ is the volume of TCA used for extraction (mL), V_sample_ is the volume of extract used for reaction (mL), V_TBA_ is the volume of TBA solution added (mL), and W is the fresh weight of the plant sample (g). The calculated MDA content was subsequently converted to nmol g^−1^ FW (1 µmol = 1000 nmol) for the presentation of the results.

### 3.5. Quantification of Total Phenolic Content

The total phenolic content (TPC) was determined daily during the first experiment. In the second experiment, the TPC was determined after 4 weeks of drought treatment and again when soil moisture reached 8–10% GWC. The TPC was evaluated using a modified Folin–Ciocalteu method [51]. For total TPC analysis, whole aboveground shoots were dried at 50 °C for 5 days. Briefly, 0.1 g of dried plant material was extracted with 10 mL of 95% ethanol and incubated for 12 h. Subsequently, 0.2 mL of the extract was mixed with 1 mL of 10% Folin–Ciocalteu reagent and thoroughly vortexed. The mixture was incubated at room temperature for 8 min, followed by the addition of 0.8 mL of 20% sodium carbonate (Na_2_CO_3_). The reaction mixture was then incubated in the dark at room temperature for 90 min. Absorbance was measured at 765 nm using a spectrophotometer. The TPC was quantified using a gallic acid calibration curve and expressed as milligrams of gallic acid equivalents per gram of dry weight (mg GAE g^−1^ DW).

### 3.6. Anatomical Analysis

In Experiment 2, anatomical characteristics were evaluated after 4 weeks of drought stress in plants maintained at 20–30% GWC (W4 treatment) and compared with the unacclimated control (W0 treatment). Samples of stems and leaves were collected for anatomical analysis and prepared as permanent sections, adapting the method in [52]. The samples were initially fixed in FAA solution for at least 24 h. The specimens were then trimmed in distilled water and post-fixed in 2.5% glutaraldehyde prepared in 0.1 M of sodium phosphate buffer (pH 7.2) for 24 h, followed by three washes with 0.1 M of sodium phosphate buffer (pH 7.2).

The samples were subsequently post-fixed in 2% osmium tetroxide for 2 h and washed three times with distilled water. Dehydration was performed using a graded acetone series (20%, 40%, 60%, and 80%), followed by three changes of 100% acetone, with each step performed for 15 min. The dehydrated samples were infiltrated with Spurr’s resin by gradually increasing the acetone-to-resin ratio to 75:25, 50:50, 25:75, and 0:100 (*v*/*v*), with each step performed for 30 min. The samples were then embedded in molds and polymerized at 80 °C.

Semi-thin sections (1 μm thick) were prepared using an ultramicrotome (EM UC7, Leica, Vienna, Austria). For staining, the sections were transferred onto glass slides containing a drop of distilled water and placed on a hot plate until the water evaporated. The sections were stained with 1% toluidine blue for 5 min and subsequently rinsed with distilled water. The slides were then placed on a hot plate until the sections became clear and were mounted as permanent preparations. Anatomical characteristics were examined and photographed using a compound light microscope (Axioscope 5, Carl Zeiss, Jena, Germany).

### 3.7. Statistical Analysis

Data were analyzed using a one-way ANOVA in a Completely Randomized Design (CRD). Comparisons of the data obtained from the different treatments were carried out based on Tukey’s multiple comparison test, with the significance level set at *p* < 0.05, using IBM SPSS Statistics, version 25 (IBM Corp., Armonk, NY, USA).

## 4. Conclusions

The findings of this study indicate that progressive water deficit stimulates an integrated response across morphological, physiological, and anatomical adaptations in water hyssop. Morphologically, reduced branching and new leaf production may have limited the development of additional transpiring surface areas, while the main shoot length and leaf size were maintained, suggesting a balance between restricting water loss and sustaining shoot growth. Short-term water limitation (11 days at 9.06% GWC) coincided with peak total phenolic content (TPC), reaching 19.57 mg GAE g^−1^ DW. Initial physiological stress was detected at 21–22% GWC, whereas 8–9% GWC marked a critical physiological threshold characterized by a pronounced reduction in photosynthetic performance and elevated oxidative stress markers. Prolonged water limitation (4 weeks at 20–30% GWC) was associated with changes in relative water content (RWC), leaf greenness, and photosynthetic performance. These changes were accompanied by anatomical modifications, including increased intercellular spaces in the leaf mesophyll, reduced intercellular spaces in the stem cortex, altered vascular bundle morphology, reduced chloroplast abundance, smaller stem xylem vessels, and increased starch accumulation in the cortex. These findings show that water hyssop exhibits coordinated morphological, physiological, and anatomical adjustments under drought stress.

## Data Availability

All relevant data supporting the findings are included within the article.

## References

[1] Kishore K., Singh M. (2005). Effect of bacosides, alcoholic extract of *Bacopa monniera* Linn. (Brahmi), an experimental amnesia in mice. Indian J. Exp. Biol..

[2] Russo A., Borrelli F. (2005). *Bacopa monniera*, a reputed nootropic plant: An overview. Phytomedicine.

[3] Saran P.L., Singh S., Solanki V.H., Kansara R.V., Damor H.I., Lodaya D.H., Kalariya K.A., Choudhary R. (2022). Impact of Partial Shades on *Bacopa monnieri* L. for Nootropic Parameters. Curr. J. Appl. Sci. Technol..

[4] Sudhakaran M.V. (2020). Botanical Pharmacognosy of *Bacopa monnieri* (Linn.) Pennell. Pharmacogn. J..

[5] Deepak M., Amit A. (2013). ‘Bacoside B’—The need remains for establishing identity. Fitoterapia.

[6] Saesong T., Temkitthawon P., Nangngam P., Ingkaninan K. (2019). Pharmacognostic and physico-chemical investigations of the aerial part of *Bacopa monnieri* (L.) Wettst. Songklanakarin J. Sci. Technol..

[7] Sosa M.D.L.M., Moroni P., O’Leary N. (2018). A taxonomic revision of the genus *Bacopa* (Gratioleae, Plantaginaceae) in Argentina. Phytotaxa.

[8] Olajide A.T., Aunsorn S., Kehinde S.A., Yazhen S., Kaewmanee T., Chusri S. (2026). Phytochemical Profile, Antioxidant Activity, and Neuroprotective Effects of *Bacopa monnieri* Extract in a Lipopolysaccharide-Induced Dementia Model. Int. J. Mol. Sci..

[9] Singh V., Jain N., Shri R. (2021). Effect of abiotic factors on bacoside A content, acetylcholinesterase inhibitory and antioxidant activities of *Bacopa monnieri* (L.) Wettst. J. Appl. Pharm. Sci..

[10] Muszyńska B., Łojewski M., Sułkowska-Ziaja K., Szewczyk A., Gdula-Argasińska J., Hałaszuk P. (2016). In vitro cultures of *Bacopa monnieri* and an analysis of selected groups of biologically active metabolites in their biomass. Pharm. Biol..

[11] Nunya N. (2019). Production of Active Ingredients in Brahmi (*Bacopa monnieri* (L.) Wettst.) under Different Cultivation Methods. Master’s Thesis.

[12] Belhassen E. (1997). Drought Tolerance in Higher Plants: Genetical, Physiological and Molecular Biological Analysis.

[13] Caser M., Chitarra W., D’Angiolillo F., Perrone I., Demasi S., Lovisolo C., Pistelli L., Pistelli L., Scariot V. (2019). Drought stress adaptation modulates plant secondary metabolite production in *Salvia dolomitica* Codd. Ind. Crops Prod..

[14] Kleinwächter M., Selmar D. (2015). New insights explain that drought stress enhances the quality of spice and medicinal plants: Potential applications. Agron. Sustain. Dev..

[15] Afroz N., Baraik B., Lal S. (2020). Effect of PEG induced drought stress on *Bacopa monnieri* (L.) Wettst. and *Adhatoda vasica* Nees. Int. J. Bot. Stud..

[16] Griesser M., Weingart G., Schoedl-Hummel K., Neumann N., Becker M., Varmuza K., Liebner F., Schuhmacher R., Forneck A. (2015). Severe drought stress is affecting selected primary metabolites, polyphenols, and volatile metabolites in grapevine leaves (*Vitis vinifera* cv. Pinot noir). Plant Physiol. Biochem..

[17] Chaves M.M., Flexas J., Pinheiro C. (2009). Photosynthesis under drought and salt stress: Regulation mechanisms from whole plant to cell. Ann. Bot..

[18] Sun Y., Wang C., Chen H.Y., Ruan H. (2020). Response of plants to water stress: A meta-analysis. Front. Plant Sci..

[19] Miller G., Suzuki N., Ciftci-Yilmaz S., Mittler R. (2010). Reactive oxygen species homeostasis and signalling during drought and salinity stresses. Plant Cell Environ..

[20] Choudhury F.K., Rivero R.M., Blumwald E., Mittler R. (2017). Reactive oxygen species, abiotic stress and stress combination. Plant J..

[21] Jogawat A., Yadav B., Chhaya, Lakra N., Singh A.K., Narayan O.P. (2021). Crosstalk between phytohormones and secondary metabolites in the drought stress tolerance of crop plants: A review. Physiol. Plant..

[22] Albergaria E.T., Oliveira A.F.M., Albuquerque U.P. (2020). The effect of water deficit stress on the composition of phenolic compounds in medicinal plants. S. Afr. J. Bot..

[23] Shin Y.K., Bhandari S.R., Jo J.S., Song J.W., Lee J.G. (2021). Effect of Drought Stress on Chlorophyll Fluorescence Parameters, Phytochemical Contents, and Antioxidant Activities in Lettuce Seedlings. Horticulturae.

[24] Edziri H., Chehab H., Aissaoui F., Boujnah D., Mastouri M. (2021). Photosynthetic, anatomical and biochemical responses of olive tree (*Olea europaea*) cultivars under water stress. Plant Biosyst..

[25] Arief M.A.A., Kim H., Kurniawan H., Nugroho A.P., Kim T., Cho B.K. (2023). Chlorophyll fluorescence imaging for early detection of drought and heat stress in strawberry plants. Plants.

[26] Das K., Roychoudhury A. (2014). Reactive oxygen species (ROS) and response of antioxidants as ROS-scavengers during environmental stress in plants. Front. Environ. Sci..

[27] Shim Y., Kim B., Choi Y., Cho S., Kim Y., Kim S., Yim Y., Kang K., Paek N. (2024). Rice OsDof12 enhances tolerance to drought stress by activating the phenylpropanoid pathway. Plant J..

[28] Salami M., Heidari B., Batley J., Wang J., Tan X.-L., Richards C., Tan H. (2024). Integration of genome-wide association studies, metabolomics, and transcriptomics reveals phenolic acid- and flavonoid-associated genes and their regulatory elements under drought stress in rapeseed flowers. Front. Plant Sci..

[29] Susilo E., Setyowati N., Parwito P., Pujiwati H. (2025). The Effect of Drought Stress on Flavonoid and Phenolic Content in Super 1 Sorghum Extract. Int. J. Integr. Sci..

[30] Basu S., Ramegowda V., Kumar A., Pereira A. (2016). Plant Adaptation to Drought Stress. F1000Research.

[31] Wahab A., Abdi G., Saleem M.H., Ali B., Ullah S., Shah W., Mumtaz S., Yasin G., Muresan C.C., Marc R.A. (2022). Plants’ Physio-Biochemical and Phyto-Hormonal Responses to Alleviate the Adverse Effects of Drought Stress: A Comprehensive Review. Plants.

[32] Domagalska M.A., Leyser O. (2011). Signal integration in the control of shoot branching. Nat. Rev. Mol. Cell Biol..

[33] Müller D., Leyser O. (2011). Auxin, cytokinin and the control of shoot branching. Ann. Bot..

[34] Baker N.R. (2008). Chlorophyll fluorescence: A probe of photosynthesis in vivo. Annu. Rev. Plant Biol..

[35] Souza R.P., Machado E.C., Silva J.A.B., Lagoa A.M.M.A., Silveira J.A.G. (2004). Photosynthetic gas exchange, chlorophyll fluorescence and some associated metabolic changes in cowpea (*Vigna unguiculata*) during water stress and recovery. Environ. Exp. Bot..

[36] Ding Y., Fromm M., Avramova Z. (2012). Multiple exposures to drought ‘train’ transcriptional responses in *Arabidopsis*. Nat. Commun..

[37] Hilker M., Schwachtje J., Baier M., Balazadeh S., Bäurle I., Geiselhardt S., Hincha D.K., Kunze R., Mueller-Roeber B., Rillig M.C. (2016). Priming and memory of stress responses in organisms lacking a nervous system. Biol. Rev..

[38] Lämke J., Bäurle I. (2017). Epigenetic and chromatin-based mechanisms in environmental stress adaptation and stress memory in plants. Genome Biol..

[39] Banik P., Zeng W., Tai H., Bizimungu B., Tanino K. (2016). Effects of drought acclimation on drought stress resistance in potato (*Solanum tuberosum* L.) genotypes. Environ. Exp. Bot..

[40] Yadav B., Jogawat A., Rahman M.S., Narayan O.P. (2021). Secondary metabolites in the drought stress tolerance of crop plants: A review. Gene Rep..

[41] Bahrami F., Arzani A., Amini H. (2013). Leaf anatomical characteristics in safflower genotypes as affected by drought stress. Acta Biol. Szeged..

[42] Shatil-Cohen A., Attia Z., Moshelion M. (2011). Bundle-sheath cell regulation of xylem-mesophyll water transport via aquaporins under drought stress: A target of xylem-borne ABA?. Plant J..

[43] Zahra N., Hafeez M.B., Kausar A., Al Zeidi M., Asekova S., Siddique K.H.M., Farooq M. (2023). Plant photosynthetic responses under drought stress: Effects and management. J. Agron. Crop Sci..

[44] Razi K., Muneer S. (2021). Drought stress-induced physiological mechanisms, signaling pathways and molecular response of chloroplasts in common vegetable crops. Crit. Rev. Biotechnol..

[45] Qaderi M.M., Martel A.B., Dixon S.L. (2019). Environmental Factors Influence Plant Vascular System and Water Regulation. Plants.

[46] Venturas M.D., Sperry J.S., Hacke U.G. (2017). Plant xylem hydraulics: What we understand, current research, and future challenges. J. Integr. Plant Biol..

[47] Lens F., Gleason S.M., Bortolami G., Brodersen C., Delzon S., Jansen S. (2022). Functional xylem characteristics associated with drought-induced embolism in angiosperms. New Phytol..

[48] Zamora Re M.I., Peplin T.M., Tomasek A. (2026). Soil Moisture Monitoring to Support Irrigation Scheduling.

[49] González L., González-Vilar M., Reigosa Roger M.J. (2001). Determination of Relative Water Content. Handbook of Plant Ecophysiology Techniques.

[50] Velikova V., Yordanov I., Edreva A. (2000). Oxidative stress and some antioxidant systems in acid rain-treated bean plants: Protective role of exogenous polyamines. Plant Sci..

[51] Singleton V.L., Orthofer R., Lamuela-Raventós R.M. (1999). Analysis of total phenols and other oxidation substrates and antioxidants by means of Folin-Ciocalteu reagent. Methods in Enzymology.

[52] Meng F., Zhang T., Yin D. (2023). The Effects of Soil Drought Stress on Growth Characteristics, Root System, and Tissue Anatomy of *Pinus sylvestris* var. *mongolica*. PeerJ.

